# Supplementary material for: Deep learning from phylogenies to uncover the epidemiological dynamics of outbreaks
Source: Nat Commun. 2022 Jul 6;13:3896. doi: 10.1038/s41467-022-31511-0 (PMC9258765; doi:10.1038/s41467-022-31511-0)
Supplement: Supplementary file 1 — Supplementary Information [file 41467_2022_31511_MOESM1_ESM.pdf]

DEEP LEARNING FROM PHYLOGENIES TO UNCOVER THE EPIDEMIOLOGICAL DYNAMICS OF OUTBREAKS

Voznica J*, Zhukova A*, Boskova V, Saulnier E, Lemoine F, Moslonka-Lefebvre M, Gascuel O*

*Correspondence: voznica.jakub@gmail.com, anna.zhukova@pasteur.fr, olivier.gascuel@mnhn.fr

SUPPLEMENTARY TABLES

	Page
**Supplementary Table 1: Method comparison in terms of accuracy**	**1**
**Supplementary Table 2: Method comparison in terms of bias**	**2**
**Supplementary Table 3: Method comparison in terms of likelihood with BD model**	**3**
**Supplementary Table 4: Parameter ranges used for simulations**	**4**
**Supplementary Table 5: Method comparison in terms of model selection**	**5**
**Supplementary Table 6: BEAST2 parameters and priors**	**6**
**Supplementary Table 7: Confidence interval assessment**	**7**

SUPPLEMENTARY FIGURES

**Supplementary Figure 1: Bijectivity of the CBLV representation**	**8**
**Supplementary Figure 2: Assessment of deep learning accuracy on small trees**	**9**
**Supplementary Figure 3 Accuracy of deep learning methods increases with tree size**	**10**
**Supplementary Figure 4: Assessment of deep learning generalization capabilities**	**11**
**Supplementary Figure 5: CNN performs well with CBLV tree representation**	**12**
**Supplementary Figure 6: How much and how fast FFNN-SS and CNN-CBLV learn?**	**13**
**Supplementary Figure 7: Adding new SS to increase accuracy of FFNN-SS**	**15**
**Supplementary Figure 8: *A priori* and *a posteriori* checks of model adequacy for HIV data**	**16**

METHODS – EXTENDED VERSION **17-37**

Supplementary Table 1: Method comparison in terms of accuracy

Model	Parameter	Mean Relative Error							
		1. FFNN SS	2. CNN CBLV	3. BEAST2	4. FFNN CBLV	5. LR SS	6. Null model 1	7. Null model 2	z-test (<0.05)
**BD 200-500 tips**	R_0	**0.07**	**0.07**	**0.06**	**0.08**	0.19	0.36	0.56	1=2=3=4
	$1/\gamma$	**0.06**	**0.06**	**0.06**	**0.06**	0.22	0.30	0.82	1=2=3=4
**BDEI 200-500 tips**	R_0	**0.08** (0.08)	**0.09** (0.09)	**0.10** (0.10)	**0.10** (0.10)	0.20 (0.20)	0.43 (0.43)	0.56	1=2=3=4
	$1/\varepsilon$	**0.13** (0.13)	**0.13** (0.13)	0.20 (0.19)	0.29 (0.29)	0.21 (0.21)	0.74 (0.74)	2.08	1=2
	$1/\gamma$	**0.10** (0.10)	**0.10** (0.10)	0.31 (0.30)	0.20 (0.21)	0.26 (0.26)	0.51 (0.51)	0.82	1=2
**BDSS 200-500 tips**	R_0	**0.09** (0.09)	**0.10** (0.10)	0.16 (0.11)	**0.10** (0.10)	0.18 (0.17)	0.34 (0.35)	0.56	1=2=4
	$1/\gamma$	**0.09** (0.09)	**0.09** (0.09)	0.22 (0.10)	**0.10** (0.10)	0.18 (0.16)	0.26 (0.27)	0.84	1=2=4
	X_{SS}	**0.11** (0.11)	**0.13** (0.13)	0.19 (0.14)	0.23 (0.22)	**0.15** (0.14)	0.27 (0.28)	0.41	1=2=5
	f_{SS}	**0.25** (0.23)	**0.26** (0.23)	0.43 (0.44)	0.33 (0.33)	0.36 (0.36)	0.34 (0.34)	0.47	1=2

For each model and inferred parameter, we compared the different inference methods in terms of Mean Relative Error (MRE). We compared these measures for 1. FFNN trained on SS, 2. CNN trained on CBLV, 3. BEAST2, 4. FFNN trained on CBLV, 5. Linear regression trained on SS, 6. "Null model 1", *i.e.* FFNN trained on SS with permuted target values and 7. "Null model 2" measured on 100 targets, where for each target we sampled randomly values from the prior parameter subspace. For 1.-6. MRE values were measured on the same 100 simulations. For BEAST2, we considered median values of covered parameter subspace, when BEAST2 did not converge (2% and 15% of simulations for BDEI and BDSS, respectively). For 1.-6. we displayed in parentheses the MRE when not considering the simulations for which BEAST2 did not converge. We compared the individual methods (1.-6.) in a pairwise fashion to find out if one was more accurate in terms of MRE than the other, using two-sided paired z-test, at significance level of 0.05 and we show the most accurate methods for each parameter in bold.

In all settings, FFNN-SS and CNN-CBLV are amongst the most accurate methods, being exclusively the most accurate ones for BDEI $1/\gamma$ and $1/\varepsilon$ and for BDSS f_{SS}. Furthermore, the comparison of MRE values shows that the prediction of f_{SS} has low accuracy for all methods (0.25 and 0.26 for FFNN-SS and CNN-CBLV, respectively, vs 0.34 obtained with "Null model 1"). This might be due to low information on superspreading individuals in the trees of this size, as supported by Supplementary Fig. 3.

Supplementary Table 2: Method comparison in terms of bias

Model	Parameter	Mean Relative Bias				
		1. FFNN SS	2. CNN CBLV	3. BEAST2	4. FFNN CBLV	5. LR SS
**BD 200-500 tips**	R_0	-0.01	-0.01	-0.01	-0.01	0.04
	$1/\gamma$	-0.01	-0.02	-0.02	-0.01	0.04
**BDEI 200-500 tips**	R_0	-0.03 (-0.03)	-0.03 (-0.03)	-0.04 (-0.04)	0.04 (0.04)	-0.03 (-0.03)
	$1/\varepsilon$	-0.01 (-0.01)	-0.01 (-0.01)	**-0.10 (-0.12)**	0.02 (0.03)	0.02 (0.02)
	$1/\gamma$	-0.03 (-0.03)	-0.01 (-0.01)	**0.24 (0.23)**	-0.03 (-0.03)	-0.01 (-0.01)
**BDSS 200-500 tips**	R_0	-0.01 (-0.01)	-0.01 (-0.01)	**0.07** (0.04)	0.00 (-0.01)	0.02 (0.00)
	$1/\gamma$	0.00 (0.00)	0.00 (-0.01)	**0.12** (0.03)	0.00 (-0.01)	0.03 (0.00)
	X_{SS}	-0.01 (-0.02)	0.01 (0.00)	**0.05** (-0.02)	**0.07** (0.04)	**0.05** (0.03)
	f_{SS}	-0.03 (-0.01)	0.02 (0.03)	**0.32 (0.36)**	-0.02 (-0.01)	**0.19 (0.20)**

For each model and inferred parameter, we compared the different inference methods in terms of Mean Relative Bias (MRB). We compared the MRB for 1. FFNN trained on SS, 2. CNN trained on CBLV, 3. BEAST2, 4. FFNN trained on CBLV and 5. Linear regression trained on SS. These MRB values were measured on the same 100 simulations. If BEAST2 did not converge, we set the inferred value to the median of covered parameter subspace. We further display MRB when not considering simulations for which BEAST2 did not converge, shown in parentheses. The relative biases bigger than 0.05 (5%) are displayed in red bold.

This shows that FFNN-SS and CNN-CBLV have low bias, while BEAST2 suffers from an intermediate to high bias with BDEI and BDSS for most parameters. These biases may account for a large fraction of MRE for BEAST2.

Supplementary Table 3: Method comparison in terms of likelihood with BD model

Y > X	Y: True values	Y: BEAST2	Y: CNN-CBLV	Y: FFNN-SS
X: True values	-	70	66	69
X: BEAST2	30	-	43	53
X: CNN-CBLV	34	57	-	55
X: FFNN-CBLV	31	47	45	-

Median Y-X	Y: True values	Y: BEAST2	Y: CNN-CBLV	Y: FFNN-SS
X: True values	0.0	4.9	4.4	4.5
X: BEAST2	-	0.0	-0.2	0.2
X: CNN-CBLV	-	-	0.0	0.2
X: FFNN-CBLV	-	-	-	0.0

For BD model and 100 large test trees (200-500 tips), we compared parameter estimates obtained with different inference methods in terms of likelihood. We compared loglikelihood values evaluated by TreePar package for: True values with which test trees were simulated; estimates obtained with BEAST2; estimates obtained with CNN-CBLV; estimates obtained with FFNN-SS. The first table shows a simple pairwise comparison (*e.g.*, 30 means that the True value was better than BEAST2 in 30% of cases), while the second table shows the median of differences between loglikelihood values.

The two results go in the same direction. The likelihood of both FFNN-SS and CNN-CBLV estimates is similar to BEAST2's, which explains the similar accuracy of the three methods (**Fig. 3**). Regarding the comparison with 'True values', if a given method tends to produce higher likelihood than that of the true parameter values, then it performs well in terms of likelihood optimization, as optimizing further should not result in higher accuracy. The results are again quite positive, as BEAST2 as well as our NNs achieved a higher likelihood than the true parameter values for ~70% of the trees, with a significant mean difference.

Supplementary Table 4: Parameter ranges used for simulations

Parameters	Name	Range	Inferred/set	Relation to other parameters
R_0	basic reproduction number	U(1,5)	inferred	$= \beta/\gamma$ (BD & BDEI)<br>$= (\beta_{S,S} + \beta_{N,N})/\gamma$ (BDSS)
$1/\gamma$	infectious period	U(1,10)	inferred	
t	tree size	"small" trees: U(50, 199);<br>"large" trees: U(200, 500)	set	
s	sampling probability	U(0.01,1)	set	
f_I	incubation factor	U(0.2,5)	parameterization	$= \varepsilon/\gamma$
$1/\varepsilon$	incubation period	[0.2, 50]	inferred	$= 1/(f_I{*}\gamma)$
X_{SS}	superspreading infectious ratio at equilibrium	U(3,10)	inferred	$= \beta_{S,S}/\beta_{N,S} = \beta_{S,N}/\beta_{N,N}$
f_{SS}	fraction of superspreading individuals at equilibrium	U(0.05, 0.20)	inferred	$= \beta_{S,S}/(\beta_{S,S}+\beta_{S,N})$<br>$= 1-\beta_{N,N}/(\beta_{N,N}+\beta_{N,S})$

For each parameter, we display its full name and the parameter range covered by simulations of training and testing sets. Note that all parameters were sampled from a uniform distribution, which we denote by U(x,y), with x being the lower and y the upper bound of that uniform distribution. The table further shows whether these parameters were inferred or used as input and, where appropriate, their relation to other model parameters (displayed in Figure 1). Parameters common to all three models are coloured in yellow, BDEI-specific in purple and BDSS-specific in green. The models are parameterized by the parameters of epidemiological interest except for the incubation factor, which enables to set the incubation period to values that are reasonable with respect to the infectious period. More specifically, through our parameterization choices, the incubation factor spans from 20% to 500% of the value of the infectious period, making it both not negligible but also not too high when compared to infectious period.

Supplementary Table 5: Method comparison in terms of model selection

a (i)

FFNN-SS	Actually BD	Actually BDEI
Predicted BD	94 (9360)	13 (1837)
Predicted BDEI	6 (640)	87 (8163)

a (ii)

FFNN-SS	Actually BD	Actually BDEI	Actually BDSS
Predicted BD	93 (9488)	11 (1006)	4 (561)
Predicted BDEI	6 (304)	86 (8783)	0 (281)
Predicted BDSS	1 (208)	3 (211)	96 (9158)

b (i)

CNN-CBLV	Actually BD	Actually BDEI
Predicted BD	96 (9289)	17 (1970)
Predicted BDEI	4 (711)	83 (8030)

b (ii)

CNN-CBLV	Actually BD	Actually BDEI	Actually BDSS
Predicted BD	91 (9131)	10 (1052)	3 (514)
Predicted BDEI	7 (519)	88 (8781)	1 (137)
Predicted BDSS	2 (350)	2 (167)	96 (9349)

c (i)

BEAST2	Actually BD	Actually BDEI
Predicted BD	75	4
Predicted BDEI	21	91
ESS<200	4	5

c (ii)

BEAST2	Actually BD	Actually BDEI	Actually BDSS
Predicted BD	62	0	2
Predicted BDEI	6	72	0
Predicted BDSS	6	7	72
ESS<200	26	21	26

Confusion matrices obtained with **a (i-ii)**, FFNN-SS, **b (i-ii)**, CNN-CBLV and **c (i-ii),** BEAST2 using AICM, either with **a-c (i)**, small trees (between 50 and 199 tips) or **a-c (ii)**, large trees (between 200 and 500 tips). BDSS and BDEI are nested within BD, namely, BDEI becomes BD when incubation period is 0 and BDSS becomes BD when superspreading individuals transmit at the same rate as normal individuals. We display the results as confusion matrices, actual classes being columns and predicted ones being rows. These were obtained with a test set of 100 simulations obtained with each model (or 10,000 for FFNN-SS and CNN-CBLV, results in parentheses). For BEAST2, 76% simulations converged for large trees and 96% for small trees. We show in red the number of simulations that did not reach an ESS of 200 for at least one parameter.

Supplementary Table 6: BEAST2 parameters and priors

Parameters	Name	BEAST2 parameter	Range	Initial value	Formula
γ	become uninfectious rate	Yes	U(0.1,1.0)	0.55	
1/γ	infectious period	No	[1,10]	1.8	
s	sampling probability	Yes	fixed	true value	
R_0	basic reproduction number	Yes	U(1.0,5.0)	3.0	$= \beta/\gamma$ (BD & BDEI)
ε	incubation rate	Yes	U(0.02,5.0)	2.51	
1/ε	incubation period	No	[0.2,50]	0.40	
$R_{0,SS}$	partial, within deme superspreading R_0	Yes	U(0.14,4.31)	1.25	
$R_{0,NN}$	partial, within deme normal spreading R_0	Yes	U(0.14,4.31)	1.44	
$R_{0,SN}$	partial, outside deme superspreading R_0	Yes	U(0.034,32.30)	9.0	
$R_{0,NS}$	partial, outside deme normal spreading R_0	Yes	U(0.034,32.30)	0.20	
R_0	basic reproduction number	No	[0.28,8.62]	2.69	$= R_{0,SS} + R_{0,NN}$ (BDSS)
X_{SS}	superspreading infectious ratio at equilibrium	No	$[4*10^{-4},127]$	6.25	$= R_{0,SS}/R_{0,NS} = R_{0,SN}/R_{0,NN}$
f_{SS}	fraction of superspreading individuals at equilibrium	No	$[4*10^{-3},0.99]$	0.12	$= R_{0,SS}/(R_{0,SS}+R_{0,SN})$<br>$= 1-R_{0,NN}/(R_{0,NN}+R_{0,NS})$

This table shows parameters and their prior distributions used during inference with BEAST2. We display the parameters, their definitions and priors in BEAST2, that are common to all models (in yellow), common to BD and BDEI (in red), BDEI-specific (in purple) and BDSS-specific (in green). From these parameters, we deduce the values and distributions of parameters of interest as shown in the table. Note that the parameters of epidemiological interest are basic reproduction number and infectious period for BD, BDEI and BDSS, incubation period for BDEI, and superspreading infectious ratio and fraction of superspreading individuals for BDSS. We check convergence (ESS) on all parameters and extract median *a posteriori* and CI values exclusively for the parameters of epidemiological interest.

Supplementary Table 7: Confidence interval assessment

Model	Parameter	Range	95% CI width and coverage					
			FFNN-SS computed on converged simulations		CNN-CBLV computed on converged simulations		BEAST2 computed on converged simulations	
			Coverage	Width	Coverage	Width	Coverage	Width
BD 200-500 tips	R_0	U(1, 5)	0.99 (0.93)	0.99 (1.0)	0.98 (0.93)	1.0 (1.1)	0.99	0.99
	1/γ	U(1, 10)	0.97 (0.92)	1.6 (1.5)	0.97 (0.92)	1.6 (1.6)	1.00	1.6
BDEI 200-500 tips	R_0	U(1, 5)	0.89 (0.93)	1.0 (1.1)	0.92 (0.93)	1.1 (1.1)	0.88	1.0
	1/ε	[0.2, 50]	0.89 (0.91)	6.6 (6.6)	0.88 (0.92)	7.1 (7.2)	0.84	6.0
	1/γ	U(1, 10)	0.84 (0.93)	2.1 (2.0)	0.93 (0.93)	2.1 (2.1)	0.90	2.1
BDSS 200-500 tips	R_0	U(1, 5)	0.94 (0.93)	1.1 (1.2)	0.93 (0.93)	1.2 (1.2)	0.94	1.1
	1/γ	U(1, 10)	0.87 (0.92)	1.6 (1.7)	0.88 (0.91)	1.6 (1.7)	0.94	1.9
	X_{SS}	U(3, 10)	0.91 (0.90)	3.5 (3.5)	0.91 (0.90)	3.6 (3.6)	0.97	3.5
	f_{SS}	U(0.05, 0.20)	0.82 (0.78)	0.087 (0.086)	0.80 (0.78)	0.089 (0.089)	0.94	0.110
BD 50-199 tips	R_0	U(1, 5)	0.90 (0.91)	1.4 (1.5)	0.90 (0.90)	1.4 (1.5)	0.91	1.4
	1/γ	U(1, 10)	0.86 (0.87)	2.3 (2.2)	0.85 (0.87)	2.3 (2.2)	0.94	2.4
BDEI 50-199 tips	R_0	U(1, 5)	0.93 (0.89)	1.6 (1.5)	0.93 (0.89)	1.6 (1.6)	0.96	1.7
	1/ε	[0.2, 50]	0.91 (0.89)	9.7 (9.3)	0.88 (0.88)	10 (9.8)	0.96	12
	1/γ	U(1, 10)	0.95 (0.90)	2.8 (2.8)	0.93 (0.90)	2.9 (2.8)	0.99	3.1

For each model and inferred parameter, we compared the different inference methods in terms of 95% confidence interval (95% CI) width and coverage, the latter being defined as the fraction of samples where the true value was within the 95% CI. We compared FFNN-SS and CNN-CBLV methods to BEAST2 on a set of 100 simulations. We did not consider the simulations for which BEAST2 did not converged (2% for BDEI large trees, 5% for BDEI small trees and 15% for BDSS large trees). We evaluated the same metrics on 10,000 simulations for FFNN-SS and CNN-CBLV (in parentheses). For FFNN-SS and CNN-CBLV we performed an approximated parametric bootstrap, while for BEAST2, we considered the entire chain with exception of the initial 10% burn-in. We highlight poor performance (coverage ≤ 0.85, width ≥ 1/3rd of prior) in one of these metrics in red and good performance (coverage ≥ 0.95) in green. Globally, the 95% CI width and coverage of FFNN-SS and CNN-CBLV are comparable with BEAST2 (while not penalizing BEAST2 for non-converged estimations) and reflect the accuracy of parameter predictions (Fig. 3 and Supplementary Fig. 2). They are slightly better for BDEI with large trees and slightly worse for BDEI with small trees. For details on how the 95% CIs were obtained, see Methods.

Supplementary Figure 1: Bijectivity of the CBLV representation

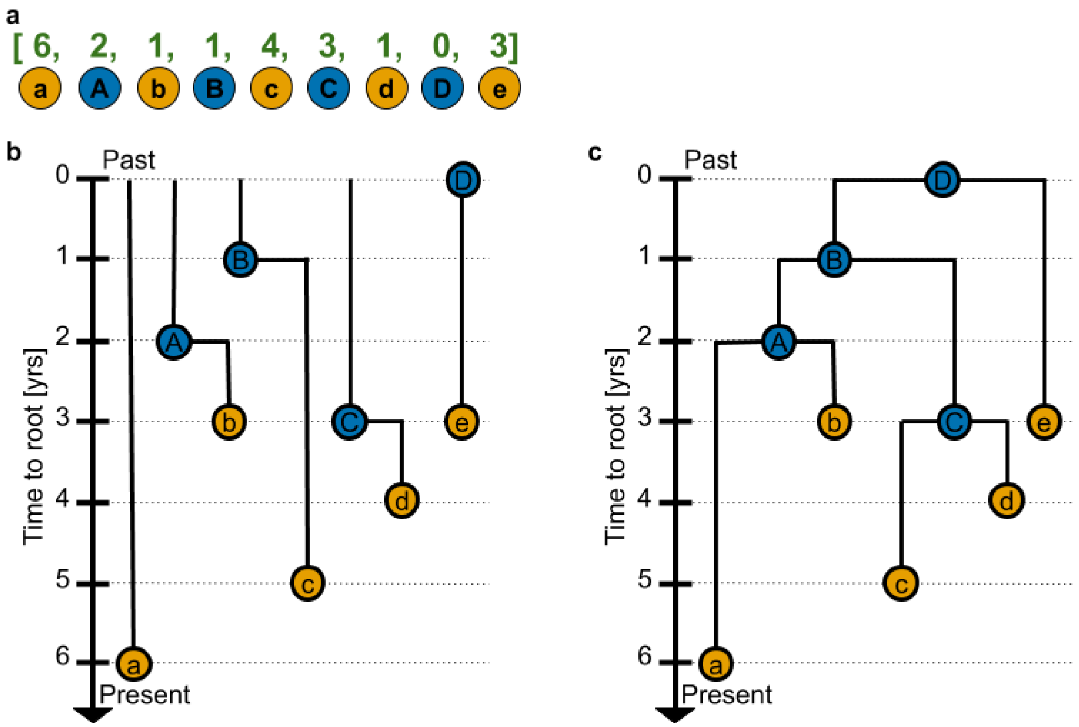

Trees are assumed to be ordered, *e.g.*, using ladderization or any other criterion. The inorder tree traversal procedure transforms an ordered tree into a unique vector. We show in this figure using a simple example, how an ordered tree is reconstructed without ambiguity from its vectorial representation, thus demonstrating the bijectivity (1-to-1 correspondence between trees and tree-compatible vectors) of the representation. **a,** shows the Compact Bijective Ladderized Vector (CBLV) representation from Figure 2 a (iii); the nodes are named alphabetically in order of appearance. Lower case letters are highlighted in yellow and represent the external nodes (or tips), upper-case letters are highlighted in blue and represent the internal nodes. **b,** depicts the creation of individual 'paths' comprising each pair of nodes: one external and one internal node, taken directly from the vector representation in the order of appearance. Note that the first external node is not paired with an internal node as it is connected to the root. **c,** shows how the tree reconstruction is achieved, by simply joining the paths from **b,** one by one from left to right. Note that not all vectors correspond to trees, as all entries must be positive or null, plus additional constraints and inequalities (*e.g.*, the second entry (A=2) must be less than (or equal to) the first entry (a=6))**.**

Supplementary Figure 2: Assessment of deep learning accuracy on small trees

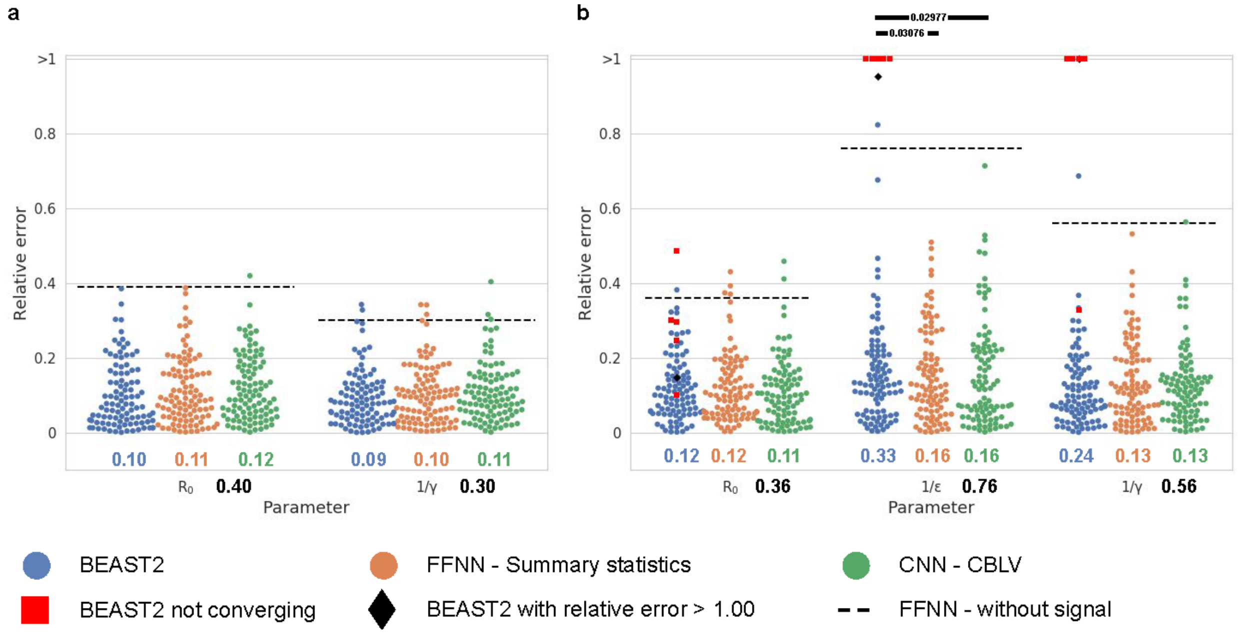

Comparison of inference accuracy by BEAST2 (in blue), FFNN-SS (in orange) and CNN-CBLV (in green) on 100 small test trees (50-199 tips). We compare the relative error for each tree, between the median *a posteriori* estimate by BEAST2 or point estimates by neural networks and the target value for each parameter. We highlight simulations for which BEAST2 did not converge and whose values were thus set to the median of the covered parameter space by depicting them as red squares. We further highlight the analyses with a high relative error (>1.00) for one of the estimates as black diamonds. We compare the relative errors for **a,** BD-simulated, and **b,** BDEI-simulated small trees. Average relative absolute error (MRE) is displayed under each distribution in the corresponding colour. The average error of an FFNN trained on summary statistics but with randomly permuted target is displayed as black dashed line and its value is shown in bold black below the x-axis. The accuracy of the output of each method is compared by two-sided paired z-test; *p-value* < 0.05 is shown as thick full line along with the corresponding *p-value*; non-significant when not shown. The accuracy is similar for the BD model, while the NNs reach better accuracy for BDEI model, while avoiding problems with convergence (5% BEAST2 inferences did not converge).

Supplementary Figure 3 Accuracy of deep learning methods increases with tree size

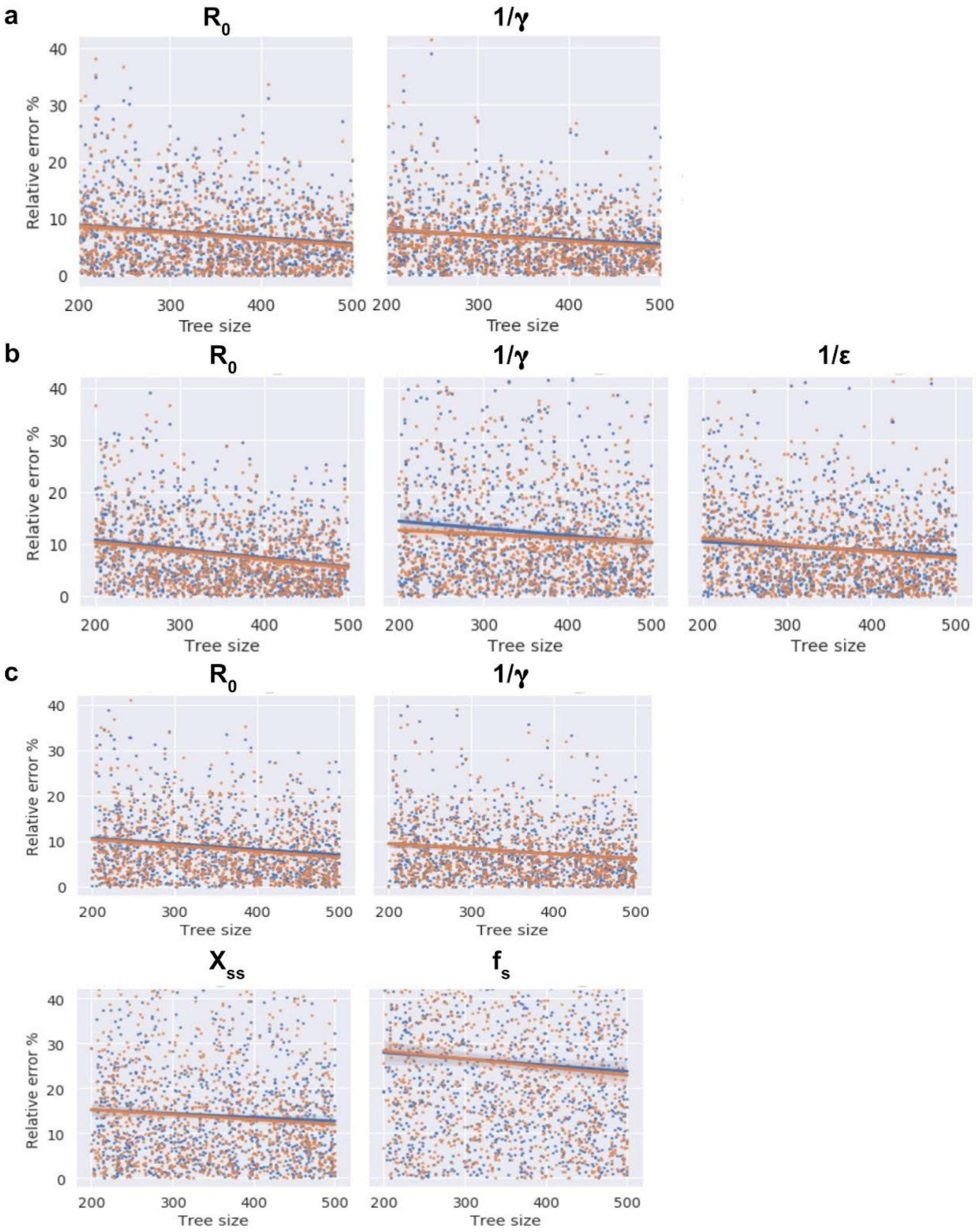

For each model **a,** BD, **b,** BDEI and **c,** BDSS, we display the regression on relative error for each parameter as a function of tree size. We show the error for both CNN-CBLV (blue) and FFNN-SS (orange) estimated for 1,000 trees (instead of 10,000 trees for display purposes). As expected, and consistent with statistical learning theory, for each parameter the accuracy increases with tree size. For example, for BDEI, the relative error of R_0 is on average 11% for trees of 200 tips and decreases to 6% for trees of 500 tips. Importantly, the relative error of superspreading fraction f_{SS}, a parameter that is difficult to estimate, decreases from 28% for trees of 200 tips to 23% for trees of 500 tips.

Supplementary Figure 4: Assessment of deep learning generalization capabilities

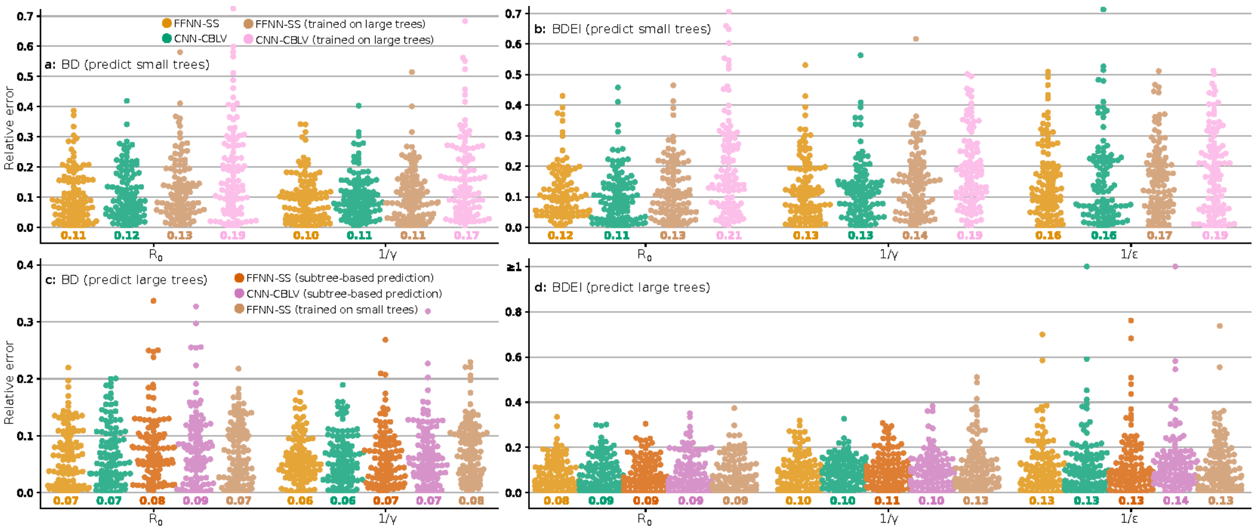

Comparison of inference accuracy by neural networks trained on trees of sizes different from those of the test trees:(top) trained on large trees and evaluated on prediction with small trees (FFNN-SS in beige, CNN-CBLV in pink); and (bottom) trained on small trees and evaluated on prediction with large trees (FFNN-SS in beige, FFNN-SS using subtree picking-and-averaging in red, CBLV-NN using subtree picking-and-averaging in magenta). For comparison, we also show FFNN-SS (orange) and CNN-CBLV (green) trained and evaluated on prediction with trees of compatible sizes (small on top, large on the bottom). The training and testing trees are the same as in **Fig. 3** (large) and **Supplementary Fig. 2** (small). We show the relative error for each test tree. The error is measured as the normalized distance between the point estimates by neural networks and the target value for each parameter. We compare the relative errors for **a, c**, BD-simulated, **b, d**, BDEI-simulated trees. Average relative error is displayed for each parameter and method in corresponding color below each figure.

The results are surprisingly good, especially with summary statistics (FFNN-SS) which are little impacted by these changes of scale as they largely rely on means. Moreover, the subtree picking-and-averaging approach performs well for both FFNN-SS and CNN-CBLV, which confirms the finding in **Fig. 4**.

Supplementary Figure 5: CNN performs well with CBLV tree representation

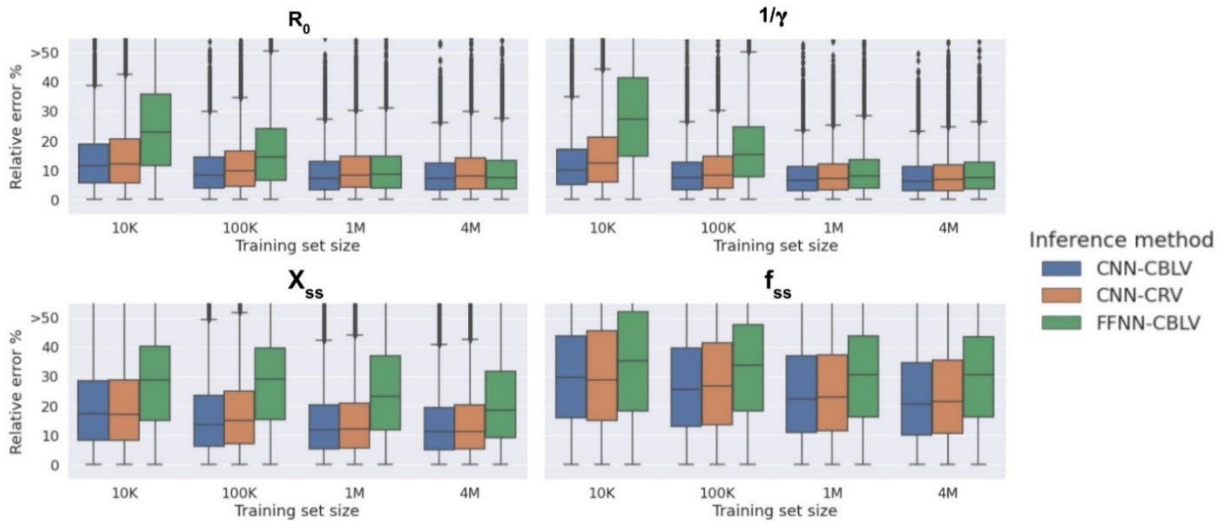

We display percentage absolute error of point estimates from deep learning methods on 10,000 test trees generated with BDSS. We compare CNN-CBLV (in blue) with FFNN-CBLV (in green) and CNN-CRV (in orange), which is a CNN trained on a Compact Random Vector (CRV) representation, where all internal nodes are randomly rotated instead of being ladderized, (in orange). In addition, we show the accuracy of these models when trained on varying training set sizes (10K: 10,000; 100K: 1,000,000; 1M: 1,000,000; and 4M: 4,000,000 trees). Boxplots are 95% CIs, the middle bar shows the mean, and the middle box corresponds to the 25% and 75% percentiles of the relative errors with 10,000 test trees.

For most parameters and training set sizes, the accuracy of CNN-CRV is lower than the one of CNN-CBLV, especially with low number of training examples, while with 4M the accuracy of both methods becomes relatively close. Thanks to ladderization, CBLV is thus enabling the CNN to learn faster and more accurately than if trained on CRV. The difference in performance is even more striking for FFNN-CBLV when compared to CNN-CBLV.CNN-CBLV is more accurate across all parameters and training set sizes, especially for X_{SS} and f_{SS} parameters even after being trained on 4M examples.

Supplementary Figure 6: How much and how fast FFNN-SS and CNN-CBLV learn?

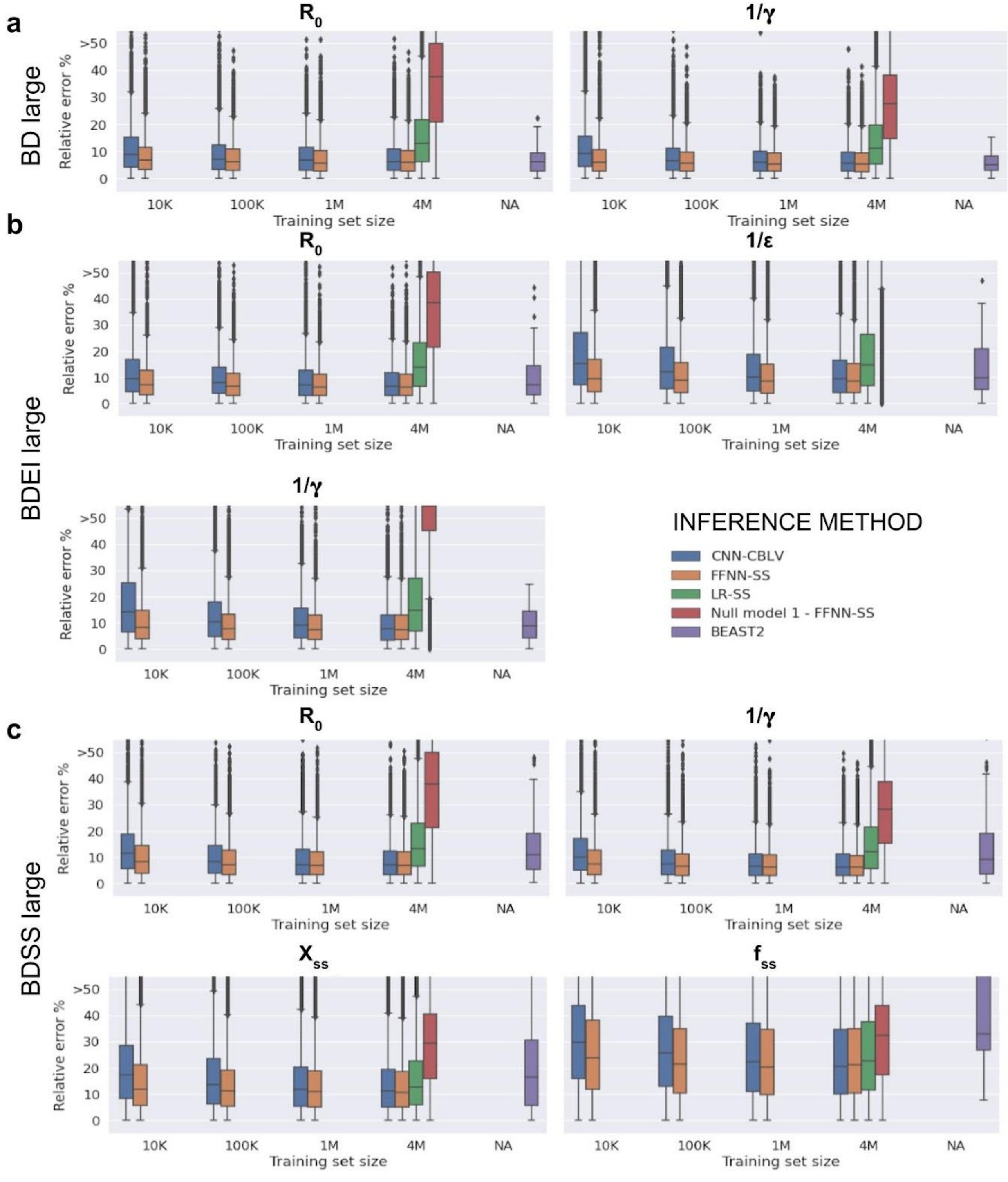

We display the distribution of relative error of individual point estimates from statistical learning methods for 10,000 trees, and median *a posteriori* values from BEAST2 for 100 large trees simulated under each of the three birth-death models **a,** BD, **b,** BDEI, **c,** BDSS. As there are many more points considered for statistical learning methods, the BEAST2 measures are shown for indicative purposes only (in purple). Boxplots are 95% CIs, the middle bar shows

the mean, and the middle box corresponds to the 25% and 75% percentiles. We compare CNN-CBLV (in blue), FFNN-SS (in orange), LR-SS as a baseline model (in green) and ”Null model 1“, for which the FFNN was trained on summary statistics to predict permuted target values (in red). The Null model maintains the same cost function as other neural networks and thus minimizes the mean percentage relative error in the absence of any signal.

In addition, we show the accuracy of estimated statistical models trained on varying training set sizes (10K: 10,000; 100K: 100,000; 1M: 1,000,000; and 4M: 4,000,000 trees), to study the efficiency of the different learning approaches.

The FFNN-SS accuracy culminates at a training size of 100,000, while CNN-CBLV keeps improving at a training size of 4,000,000. This is consistent with the fact that summary statistics represent high-level information, while the CNN has to learn how to infer parameter values from raw information and thus require many more examples.

The baseline model LR-SS does not reach the same level of accuracy as FFNN-SS and CNN-CBLV for most parameters, but X_{SS} and f_{SS} parameters in BDSS. The relationship between the summary statistics and the studied model parameter values is too complex to be handled by linear regression. Finally, by comparing the accuracies to those of “Null model 1”, we show how much information is extracted by the properly set-up deep-learning methods as opposed to a model trained in the absence of signal. In most cases, the accuracy gain is high. The only exception is f_{SS} parameter, where the “Null model 1” has an accuracy of 0.33, while with CNN-CBLV and FFNN-SS we reach an accuracy of around 0.25. These two approaches thus do not estimate this parameter very accurately, most likely because (1) f_{SS} is a fraction of rates and thus cumulates several errors, and (2) the information in the tree on superspreading is low, as the fraction and thus the number of superspreading individuals is low as well. One solution to this problem is to gather more data. Indeed, as shown in Supplementary Fig.7 accuracy increases substantially when learning and inferring on larger trees.

Supplementary Figure 7: Adding new SS to increase accuracy of FFNN-SS

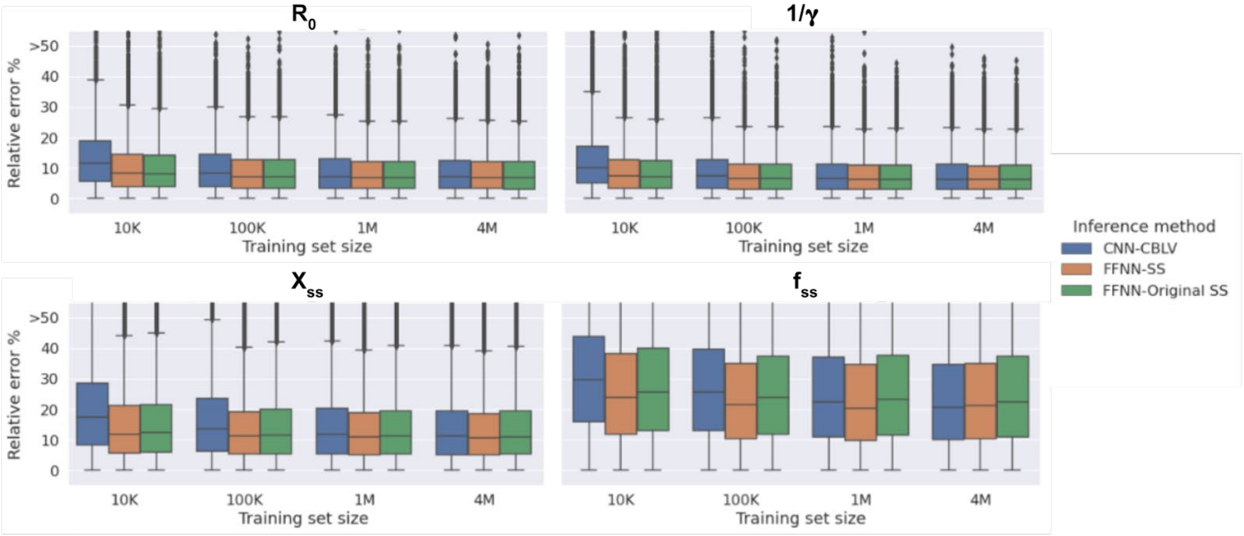

Adding SS on transmission chains improves the accuracy of prediction of superspreading individual frequency f_{ss} in the BDSS model. We display relative absolute error (RE) of point estimates from deep learning methods for 10,000 trees simulated under the BDSS model. Boxplots are 95% CIs, the middle bar shows the mean, and the middle box corresponds to the 25% and 75% percentiles. We compare CNN trained on CBLV representation with FFNN trained on SS and on Original Saulnier's SS (*i.e.,* without SS on transmission chains), for each parameter of interest. In addition, we show the accuracy of NN models trained on varying training set sizes (10K: 10,000; 100K: 100,000; 1M: 1,000,000; and 4M: 4,000,000 trees). This shows that additional SS enable to decrease the MRE by over 2% for f_{ss} making it comparable to CNN-CBLV accuracy with large training sample (4M).

Supplementary Figure 8: ***A priori*** **and** ***a posteriori*** **checks of model adequacy for HIV data**

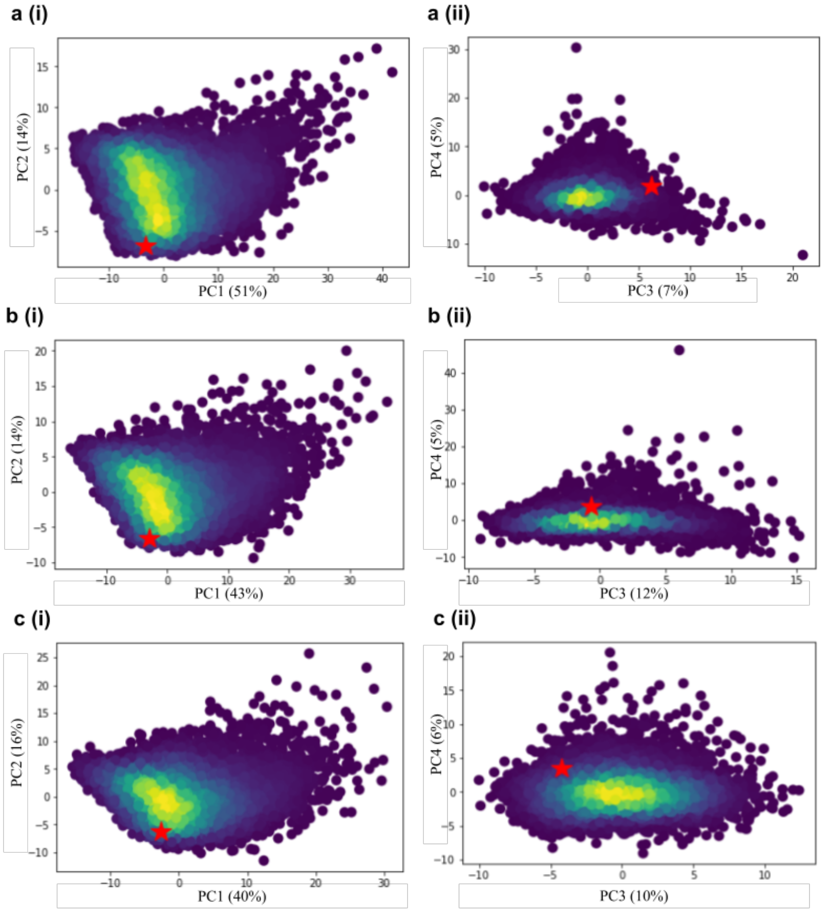

d

Model	# SS outside Min-Max	SS Names
BD	5	ie BL mean 2 and 3, ie BL median 2 and 3, ie BL var 3
BDEI	5	min Height, ie BL mean 2 and 3, ie BL median 3, ie BL var 3
BDSS	0	-

e

BDSS	# SS outside Min-Max	SS Names
FFNN-SS	13	max_L, slope_1, mean_s_time, x_16, x_17, x_18, y_3-y_9
CNN-CBLV	20	max_L, t_max_L, slope_1, mean_s_time, x_11-x_18, y_2-y_9

For each model **a**, BD, **b**, BDEI and **c**, BDSS, we encoded 10,000 simulations from the test set into SS and standardized them. We then performed principal component analysis (PCA) and projected the SS from HIV phylogeny (red star) on these PCA plots. Here we show the projections along **a-c (i)**, the 1st and the 2nd components (PC1 and PC2) and **a-c (ii)**, the 3rd and the 4th components (PC3 and PC4) of the PCA, together with the associated percentage variance explained in parentheses. For each model and projection, the HIV data point is surrounded by the simulations, meaning it resembles globally the simulations and thus we can apply our deep learning (and BEAST2) inference methods under each birth-death model.

Furthermore, we performed more detailed **d**, *a priori* and **e**, *a posteriori* checks using directly the values of summary statistics without a PCA. For each statistics of HIV phylogeny, we checked whether it lays between the minimum and the maximum value of this statistics covered in **d**, test set of 10,000 trees for given model (BD, BDEI or BDSS), **e**, the *a posteriori* set under BDSS (10.000 simulations, see text). The statistics that were outside the minimum and maximum values are numbered and named in **d** and **e**. All rejected SS in *a posteriori* check (**e**) correspond to the LTT plot (*e.g.*, x and y coordinates), consistent with the fact that the probabilistic, sampling component of the BDSS model is an oversimplification of actual sampling schemes, which depend on contact tracing, sampling campaigns and policies, etc. For details on each statistics, refer to https://doi.org/10.1371/journal.pcbi.1005416.

METHODS – EXTENDED VERSION

	Page
**TREE REPRESENTATION USING SUMMARY STATISTICS (SS)**	**19**
Saulnier et al. summary statistics	**19**
Additional summary statistics	**19**
**COMPLETE AND COMPACT TREE REPRESENTATION (CBLV)**	**20**
Tree ladderization	**20**
Tree traversal and encoding	**21**
Properties of CBLV	**21**
Alternative tree representations	**22**
**TREE RESCALING**	**22**
**REDUCTION AND CENTERING OF SUMMARY STATISTICS REPRESENTATION**	**22**
**PARAMETER INFERENCE USING NEURAL NETWORKS**	**23**
Deep feedforward neural network architecture for SS	**23**
Deep convolutional neural network for CBLV	**24**
Neural network setting and training	**24**
Preventing overfitting: Early stopping and Dropout	**24**
Neural networks for model selection	**24**
Parameter estimation from very large trees using subtree picking and averaging	**25**
**CONFIDENCE INTERVALS (95% CI)**	**26**
Computation of 95% CI	**26**
Assessment of CI accuracy and width	**27**
**MODEL ADEQUACY**	**28**
***A priori* checks**	**28**
***A posteriori* checks**	**29**

MODELS **29**

Constant rate birth-death model with incomplete sampling **29**

Birth-death model with exposed-infectious classes **29**

Birth-death model with superspreading **30**

SIMULATIONS **30**

METHOD COMPARISON **31**

Parameter inference with BEAST2 **31**

Model selection with BEAST2 **32**

Linear regression **33**

FFNN-CBLV **33**

TreePar **33**

Null models **34**

PERFORMANCE ASSESSMENT **34**

Mean relative error MRE **34**

Mean relative bias MRB **35**

Likelihood-based assessment **35**

Model selection accuracy **36**

Comparison of time efficiency **36**

HIV DATASET **37**

PHYLODEEP SOFTWARE **37**

ADDITIONAL REFERENCES **37**

TREE REPRESENTATION USING SUMMARY STATISTICS (SS)

We use a set of 98 summary statistics (SS), to which we add the sampling probability, summing to a vector of 99 values.

Saulnier et al summary statistics

We use the 83 SS proposed by Saulnier *et al.*[19]:

- 8 SS on tree topology
- 26 SS on branch lengths
- 9 SS on Lineage-Through-Time (LTT) plot
- 40 SS providing the coordinates of the LTT plot

The computing time of these statistics grows linearly with tree size. For details, see the original paper.

Additional summary statistics

In addition to Saulnier *et al.*[19] statistics, we designed 14 SS on transmission chains. Moreover, we provide the number of tips in the tree as input resulting in 83+14+1 = 98 SS in total.

The statistics on transmission chains are designed to capture information on the superspreading population. A superspreading individual transmits to more individuals within a given time period than a normal spreader. We thus expect that with superspreading individuals we would have shorter transmission chains. To have a proxy for the transmission chain length, we look at the sum of 4 subsequent shortest times of transmission for each internal node. This gives us a distribution of time-durations of 4-transmission chains. We assume that information on the transmission dynamics of superspreading individuals is retained in the lower (*i.e.*, left) tail of 4-transmission-chain lengths distribution, which contains relatively many transmissions with short time to next transmission, while the information on normal spreaders should be present in the rest of the distribution.

The implementation of these 4-transmission-chain SS is the following. For each internal node, we sum the distances from the internal node to its closest descendant nodes, descending exactly four times, that is, we take first the distance from the given internal node to its closest child node (of level 1), then from the (level 1) child node, we take its distance to its own closest child node (of level 2), *etc.* If one of the closest descendant nodes is a tip (except for the last one in

the chain), we do not retain any value for the given internal node. Other options, like the shortest 4-edge pathway, could have been used as well and would likely give comparable results.

On the obtained distribution of 4-transmission-chain lengths, we compute 14 statistics:

- number of 4-transmission chains in the tree
- 9 deciles of 4-transmission-chain lengths distribution
- minimum and maximum values of 4-transmission-chain lengths distribution
- mean value of 4-transmission-chain lengths
- variance of 4-transmission-chain lengths

Adding the same summary statistics but on chains comprising 2, 3 and 5 consecutive transmissions had a negligible impact on parameter inference accuracy (data not shown).

COMPLETE AND COMPACT TREE REPRESENTATION (CBLV)

Simulated dated trees are encoded in the form of real-valued vectors, which are then used as input for the neural networks. The representation of a tree with n tips is a vector of length $2n$-1, where one single real-valued scalar corresponds to one internal node or tip. This representation thus scales linearly with the tree size. The encoding is achieved in two steps: tree ladderization and tree traversal.

Tree ladderization

The tree ladderization consists in ordering the children of each node. Child nodes are sorted based on the sampling time of the most recently sampled tip in their subtrees: for each node, the branch supporting the most recently sampled subtree is rotated to the left, as in **Fig. 2 a (i-ii)**.

We considered several alternatives with different criteria for child (subtree) sorting instead of ladderization: sampling time of the most anciently sampled tip, subtree length (*i.e.*, sum of all branch lengths including the rooting branch), diversification (*i.e.*, number of tips), normalized branch lengths (*i.e.*, subtree length divided by the number of tips), *etc*. These did not yield better results than CBLV. We show in **Supplementary Fig. 5** the comparison of CBLV with Compact Random Vector (CRV), for which internal nodes were sorted randomly before the tree traversal, showing that CRV yields poorer results than CBLV, as expected.

Tree traversal and encoding

Once the tree is sorted, we perform an inorder tree traversal, using a standard recursive algorithm from the depth first family[30]. When visiting a tip, we add its distance to the previously visited internal node or its distance to the root, for the tip that is visited first (*i.e.*, the tree height due to ladderization). When visiting an internal node, we add its distance to the root. Examples of encoding are shown in **Fig. 2 a (ii-iii)**. This gives us the Compact Bijective Ladderized Vector (CBLV). We then separate information relative to tips and to internal nodes into two rows (**Fig. 2 a (iv)**) and complete the representation with zeros until reaching the size of the largest simulated tree for the given simulation set (**Fig. 2 a (v)**).

Properties of CBLV

CBLV has favourable features for deep learning. Ladderization does not actually change the input tree (phylogenies are unordered trees), but by ordering the subtrees it standardizes the input data and facilitates the learning phase, as observed with CRV (**Supplementary Fig. 5**). Then, the inorder tree traversal procedure is a bijective transformation, as it transforms a tree into a tree-compatible vector, from which the (ordered) tree can be reconstructed unambiguously, using a simple path-agglomeration algorithm shown in **Supplementary Fig. 1**. Note, however, that not all vectors are tree-compatible (*e.g.,* all entries must be non-negative; the second entry must be less than the first one, *etc*.). CBLV is "as concise as possible" being composed of $2n$-1 real values (**Fig. 2 a (iii)**), where n is the number of tips. A rooted tree has $2n$-2 branches, and thus $2n$-2 entries are needed to represent the branch lengths. In our $2n$-1 vectorial encoding of trees, we not only represent the branch lengths, but also the tree topology using only 1 additional entry.

The compactness and bijectivity of tree representation reduce the number of simulations required for training the neural network (**Supplementary Fig. 5**). This is because the number of parameters to be trained remains reasonable with compact representation. Moreover, the networks do not need to learn that several different inputs correspond to the same tree.

Our neural networks are intended to apply to trees of variable sizes (*e.g.*, trees of 200 to 500 tips in our experiments with 'large' trees). Thus, they are trained on representations of different lengths (*e.g.*, a vector of length 399 for a tree of 200 tips), that we complete with zeroes to reach the length of the largest trees (*i.e.*, 999 for 500 tips). We add an additional zero to obtain a two-row matrix (500*2 for 500 tips).

Alternative tree representations

Our CBLV tree representation could likely be improved to ease the learning phase and obtain even better parameter estimates. We tested several alternative representations, some inspired by the polynomial representation of small subtrees[26,27], the Laplacian spectrum[28] and additive distance matrices that are equivalent to trees[58]. None was by far as convincing as CBLV, which is likely due to their large size (*e.g.*, n^2 for distance matrices) or numerical instabilities and potential loss of information (*e.g.*, for Laplacian spectrum). Moreover, the margin for improvement of the accuracy of CNN-CBLV for the BD model, and likely for other models, is low. This is due to the observation that the accuracy of CNN-CBLV is similar to that of likelihood-based approaches for the BD model, and the fact that we have an analytical likelihood formula for the BD model, making the likelihood-based approach itself optimal[8,9].

TREE RESCALING

Before encoding, the trees are rescaled so that the average branch length is 1, that is, each branch length is divided by the average branch length of the given tree, called rescale factor. The values of the corresponding time-dependent parameters (*i.e.*, infectious period and incubation period) are divided by the rescale factor too. The NN is then trained to predict these rescaled values. After parameter prediction, the predicted parameter values are multiplied by the rescale factor and thus rescaled back to the original time scale.

This step enables us to overcome problems of arbitrary time scales of input trees and makes a pre-trained NN more generally applicable. More specifically, an input tree with a time scale in days will be associated naturally with the same output as the same tree with a time scale in years, since both these trees will be rescaled to the same intermediate tree with average branch length of 1. Rescaling thus makes it possible to apply the same pre-trained NN to phylogenies reconstructed from sequences of a pathogen associated with an infectious period on the scale of days (*e.g.*, Ebola virus) or years (*e.g.*, HIV).

REDUCTION AND CENTERING OF SUMMARY STATISTICS REPRESENTATION

Before training our NN and after having rescaled the trees to unit average branch length (see the sub-section above), we reduce and center every summary statistic by subtracting the mean and scaling to unit variance. To achieve this, we use the standard scaler from the scikit-learn package[50], which is fitted to the training set. This does not apply to CBLV representation, to avoid losing the ability to reconstruct the tree.

PARAMETER AND MODEL INFERENCE USING NEURAL NETWORKS

We implemented deep learning methods in Python 3.6 using Tensorflow 1.5.0[51], Keras 2.2.4[52] and scikit-learn 0.19.1[50] libraries. For each network, several variants in terms of number of layers and neurons, activation functions, regularization, loss functions and optimizer, were tested. In the end, we decided for two specific architectures that best fit our purpose: one deep FFNN trained on SS and one CNN trained on CBLV tree representation.

Deep feedforward neural network architecture for SS

The network consists of one input layer (of 99 input nodes both for trees with 50-199 and 200-500 tips), 4 sequential hidden layers organized in a funnel shape with 64-32-16-8 neurons and 1 output layer of size 2-4 depending on the number of parameters to be estimated. The neurons of the last hidden layer have linear activation, while others have exponential linear activation[53].

```
_________________________________________________________________
Layer (type)                 Output Shape              Param #   
=================================================================
dense_1 (Dense)              (None, 64)                6400      
_________________________________________________________________
dense_2 (Dense)              (None, 32)                2080      
_________________________________________________________________
dense_3 (Dense)              (None, 16)                528       
_________________________________________________________________
dense_4 (Dense)              (None, 8)                 136       
_________________________________________________________________
dense_5 (Dense)              (None, 2)                 18        
=================================================================
Total params: 9,162
Trainable params: 9,162
Non-trainable params: 0
```

Architecture: **Feedforward neural network architecture.** Example of FFNN trained on large trees to estimate the parameters of the BD model (R_0 and infectious period $1/\gamma$). 'Dense' layer means that for each neuron, all the inputs are multiplied by learned weights, summed together with the bias term. The activation function is then applied to the weighted sum before being output to the next layer. Dense_1 to dense_4 are layers with neurons of exponential linear activation, while dense_5 is composed either of softmax (in case of model selection) or of linear neurons (in case of parameter estimation). The number of trainable parameters in each layer is displayed (Param #): for example in the first layer, we have 99 input values and 1 bias for each of the 64 neurons, giving us in total (99+1)*64=6,400 trainable parameters. Output by Keras[52], the 'None' in the 'Output Shape' means the network can input more than one training example at the time and that there is no constraint on the batch size (hence 'None').

Deep convolutional neural network for CBLV

The CNN consists of one input layer (of 400 and 1002 input nodes for trees with 50-199 and 200-500 tips, respectively). This input is then reshaped into a matrix of size of 201*2 and 501*2, for small and large trees, respectively, with entries corresponding to tips and internal nodes separated into two different rows (and one extra column with one entry in each row corresponding to the sampling probability). Then, there are two 1D convolutional layers of 50 kernels each, of size 3 and 10, respectively, followed by max pooling of size 10 and another 1D convolutional layer of 80 kernels of size 10. After the last convolutional layer, there is a GlobalPoolingAverage1D layer and a FFNN of funnel shape (64-32-16-8 neurons) with the same architecture and setting as the NN used with SS.

Neural network setting and training

For both NNs, we use the Adam optimisation algorithm[54] as optimizer and the Mean Absolute Percentage Error (MAPE) as loss function. The batch size is set to 8,000. To train the network, we split the simulated dataset into 2 groups: (1) proper training set (3,990,000 examples); (2) validation set (10,000).

Preventing overfitting: Early stopping and Dropout

To prevent overfitting during training, we use: (1) the early stopping algorithm evaluating MAPE on a validation set; and (2) dropout that we set to 0.5 in the feed-forward part of both NNs[55] (0.4, 0.45, 0.55 and 0.6 values were tried for basic BD model without improving the accuracy).

Neural networks for model selection

For model selection, we use the same architecture for FFNN-SS and CNN-CBLV as those for parameter inference described above**.** The only differences are: (1) the cost function: categorical cross entropy and (2) the activation function used for the output layer, that is, softmax function (of size 2 for small trees, selecting between BD and BDEI model, and of size 3 for large trees, selecting between BD, BDEI and BDSS). As we use the softmax function, the outputs of prediction are the estimated probabilities of each model, summing to 1.

The FFNN-SS and CNN-CBLV are trained on $8*10^6$ trees in the small tree setting ($4*10^6$ trees per model, BD and BDEI). In the large tree setting, the FFNN-SS is trained on $12*10^6$ trees ($4*10^6$ trees per model, BD, BDEI and BDSS)

and the CNN-CBLV is trained on $9*10^6$ trees ($3*10^6$ trees per model, BD, BDEI and BDSS), instead of $12*10^6$ for GPU limitation purposes.

Parameter estimation from very large trees using subtree picking and averaging

To predict from very large trees (*e.g.*, our 'huge' trees having 5,000 to 10,000 tips, **Fig. 4**) we designed the 'Subtree Picker' algorithm. The goal of Subtree Picker is to extract subtrees of bounded size representing independent sub-epidemics within the epidemic represented by the initial huge tree T, while covering most of the initial tree branches and tips in T. The sub-epidemics should follow the same sampling scheme as the global epidemic. This means that we can stop the sampling earlier than the most recent tip in T, but we cannot omit tips sampled before the end the sampling period (this would correspond to lower sampling probability). Each picked subtree corresponds to a sub-epidemic that starts with its root individual and lasts between its root date D_{root} and some later date ($D_{last} > D_{root}$). The picked subtree corresponds to the top part of the initial tree's clade with the same root, while the tips sampled after D_{last} are pruned.

The picked subtrees do not intersect with each other and contain between *m* and *M* tips each. Together they cover most of the initial tree's branches. The initial tree T contains more than *M* tips. In the current PhyloDeep setting, *M*=500 (the largest tree size in the training set) and *m*=200 for BDSS and =50 for BD and BDEI (the smallest tree size in the training set).

Subtree Picker performs a postorder tree traversal (tips-to-root), where for each tree node N it calculates the maximum number of tips t_N that can be extracted from its subtrees. The algorithm is recursive and combines two basic strategies: (1) the subtree rooted with N is decomposed into two independent sub-epidemics corresponding to N's direct descendants; or (2) we decide to keep the upper part (with *x* oldest tips, $m \leq x \leq M$) of the subtree rooted with N, and the rest of N's descendants is decomposed into independent sub-epidemics. The recursion is as follows (size(N) is the number of tips in the subtree rooted with N):

If size(N) < *m*, then t_N=0
Else if $m \leq$ size(N) $< M + m$, then t_N=max(size(N), M) #*pick the root subtree containing* t_N *oldest tips*
Else pick the best between the two strategies:
(1) Left L and right R children of N lead to independent sub-epidemics (subtrees): $t_N = t_L + t_R$

(2) Pick the root subtree of x oldest tips ($m \leq x \leq$ M, all possible x compared) plus the set Δ of the oldest descendant nodes of N, which represents the roots of independent sub-epidemics sampled after x^{th} tip date: $t_N = x + \sum_{D \text{ in } \Delta} t_D$

For each processed node N, its optimal subtree picking strategy is memorized. Once t_N is calculated for the root of the global tree T, the algorithm picks the root's subtrees according to the chosen strategy and, if needed, descends to the non-affected descendant nodes to pick more subtrees.

Let n be the size of T (number of tips). This tree decomposition requires one preorder tree traversal, with computing time in $O(n)$. However, the picking strategy (2) requires another $O(s)$ time to extract Δ (with appropriate data structure and pre-treatments). Thus, the whole computing time (computing (2) for each node in the tree traversal) is in $O(n^2)$, in the worst case. In practice, the subtrees extracted by Subtree Picker cover on average 98.5% (BD), 97.3% (BDEI) and 82.4% (BDSS) of the initial tree branches on the 'huge' tree datasets (5,000 to 10,000 tips). For the BDSS model this percentage is lower than for BD and BDEI, because of the narrower subtree size interval (m=200, M=500 versus m=50, M=500) corresponding to current PhyloDeep training set settings. In terms of computing time, Subtree Picker takes on average 0.6 (BD), 0.8 (BDEI) and 0.8 (BDSS) seconds per 'huge' tree, meaning that it could easily be applied to much larger trees.

Once subtrees (sub-epidemics) have been extracted, they are analysed using CNN-CBLV or FFNN-SS, and the parameter estimates are averaged with weights proportional to subtree sizes (number of tips).

CONFIDENCE INTERVALS (95% CI)

Computation of 95% CI

We compute 95% CI using parametric bootstrap. To facilitate the deployment and speed-up the computation, we perform an approximation using a separate set of 1,000,000 simulations for calculation of CI. For each simulation in the CI set, we store the true parameter values *(i.e.*, values with which we simulated the tree) and the parameter values predicted with both of our methods. This large dataset of true/predicted values is used to avoid new simulations, as required with the standard parametric bootstrap.

For a given simulated or empirical tree T, we obtain a set of predicted parameter values, $\{p\}$. The CI computation procedure searches among stored data those that are closest to T in terms of tree size, sampling probability and predicted values. We first subset:

- 10% of simulations within the CI set, which are closest to T in terms of size (number of tips), thus obtaining 100,000 CI sets of true/predicted parameter values.
- Amongst these, 10% of simulations that are closest to T in terms of sampling probability.

We thus obtain 10,000 CI sets of real/predicted parameter values, similar in size and sampling probability to T. For each parameter value *p* predicted from T, we identify the 1,000 nearest neighbouring values amongst the 10,000 true values of the same parameter available in the CI sets, $R_{CI} = \{r_{i=1,1000}\}$, and keep the corresponding predicted values, $P_{CI} = \{p_{i=1,1000}\}$. We then measure the errors for these neighbours as $E_{CI} = \{e_i = p_i - r_i\}$. We center these errors around *p* using the median of errors, $m(E_{CI})$, which yields the distribution of errors for given prediction *p*: $D = \{p + e_i - m(E_{CI})\}$, from which we extract the 95% CI around *p*. Individual points in the obtained distribution that are outside of the parameter ranges covered through simulations are set to the closest boundary value of the parameter range. For example, for f_{SS}, if for a point in the distribution we obtain a value lower than 0.05, we set the value of that point to 0.05; and if we obtain a value larger than 0.20, we set it to 0.20. We undertake this procedure for all parameters except for the time related ones, that is, infectious and incubation period as these depend on the time rescaling. The width of our 95% CIs is defined as the distance between the 2.5% and 97.5% percentile. With very large trees and the subtree picking and averaging procedure, we consistently use a quadratic weighted average of the individual CIs found for every subtree.

Assessment of 95% CI coverage and width

To assess this fast implementation of the parametric bootstrap, we used the test set of 10,000 simulations (and 100 simulations for comparison with BEAST2 95% CI). We measured the coverage being defined as the fraction of simulations where the true/target parameter values are inside the obtained 95% CI:

$$95\%\text{CIaccuracy} = \frac{\#\text{ true values inside }95\%\text{CI}}{\#\text{ simulations}}$$

We applied the same criteria for BEAST2. For comparison of all methods, we excluded BDEI and BDSS simulations for which BEAST2 did not converge after 10 million steps. To draw BEAST2 CIs, we discarded the burn-in, that is, the first 10% of the MCMC, and calculated the CI on the remaining part of the chain. The CI width and coverage within the CIs obtained by NNs and BEAST2 are reported in **Supplementary Tab. 7**.

There exists a plethora of approaches for assessment of uncertainty and CI estimation. For example, (1) in a similar ABC context, the use of neighbouring trees (based on the Euclidean distance, not applicable to CBLV and questionable with SS) combined with a regression-based correction similar to that explained above[19,20]; (2) the (non-approximated) parametric bootstrap[59]; (3) the prediction of values from a distribution of trees reconstructed with Bayesian methods[10]; *etc*. We chose an approximation of the parametric bootstrap for its easy deployability, speed, coverage and width of produced CIs. The easy deployability comes from the fact that CIs are based on pre-calculated data stored in our CI set. The speed of the method comes from it not requiring simulations of new trees, and thus producing CIs within 2-4 seconds. The coverage and width are comparable to those of BEAST2 (**Supplementary Tab. 7**), a Bayesian method, intended to estimate the distribution of parameters and the uncertainty of inferences, with high computational cost.

MODEL ADEQUACY

A priori checks

We performed a sanity check using the SS of the test set simulations and the SS measured on the empirical HIV phylogeny. We reduced and centered the SS and performed a Principal Component Analysis (PCA) using the PCA function from the scikit-learn[50] package.

We highlighted the data point corresponding to the Zürich HIV MSM phylogeny in **Supplementary Fig. 8**, for each model (BD, BDEI and BDSS). Dissemblance between the simulations and the HIV phylogeny would be manifested by the fact that this data point lies outside the distribution corresponding to the simulations.

Furthermore, we performed an additional *a priori* check consisting in the study of all individual SS rather than dimensional reduction with PCA. For each SS, we checked whether the value for Zürich HIV MSM phylogeny lays between the minimum and maximum value of that SS in the test set of 10.000 trees. We reported the results in **Supplementary Fig. 8**.

A posteriori checks

We performed tests analogous to the *a priori* model adequacy checks. For both PCA and individual SS tests, instead of using the test set as representative of simulations, we simulated 10,000 additional simulations under the selected BDSS model. Parameter values were resampled from uniform distribution with boundaries given by the 95% CIs, and sampling probability fixed to presumed value of 0.25 (**Fig. 5, Supplementary Fig. 8**).

MODELS

The models we used for tree simulations are represented in the form of flow diagrams in **Fig. 1**. We simulated dated binary trees for (1) the training of NNs and (2) accuracy assessment of parameter estimation and model selection. We used the following three individual-based phylodynamic models:

Constant rate birth-death model with incomplete sampling

This model (BD[8,9], **Fig. 1 a**) contains three parameters and three compartments: infectious (I), removed with sampling (R) and removed unsampled (U) individuals. Infection takes place at rate β. Infectious individuals are removed with rate γ. Upon removal, an individual is sampled with probability *s*.

For simulations, we re-parameterized the model in terms of: basic reproduction number, R_0; infectious period, 1/γ; sampling probability, *s*; and tree size, *t*. We then sampled the values for each simulation uniformly at random in the ranges given in **Supplementary Tab. 4**.

Birth-death model with exposed-infectious classes

This model (BDEI[10-12], **Fig. 1 b**) is a BD model extended through the presence of an exposed class. More specifically, this means that each infected individual starts as non-infectious (E) and becomes infectious (I) at incubation rate ε. BDEI model thus has four parameters (β, γ, ε and *s*) and four compartments (E, I, R and U).

For simulations, we re-parameterized the model similarly as described for BD, set the ε value via 1/γ and incubation ratio (=ε/γ). We sampled all parameters, including ε/γ, from a uniform distribution, just as with BD (**Supplementary Tab. 4**).

Birth-death model with superspreading

This model (BDSS[5,10,11], **Fig. 1 c**) accounts for heterogeneous infectious classes. Infected individuals belong to one of two infectious classes (I_S for superspreading and I_N for normal spreading) and can transmit the disease by giving birth to individuals of either class, with rates $\beta_{S,S}$ and $\beta_{S,N}$ for I_S transmitting to I_S and to I_N, respectively, and $\beta_{N,S}$ and $\beta_{N,N}$ for I_N transmitting to I_S and I_N, respectively. However, there is a restriction on parameter values: $\beta_{S,S} \times \beta_{N,N} = \beta_{S,N} \times \beta_{N,S}$. There are thus superspreading transmission rates $\beta_{S,.}$ and normal transmission rates $\beta_{N,.}$ that are $X_{SS} = \beta_{S,S} / \beta_{N,S} = \beta_{S,N} / \beta_{N,N}$ times higher for superspreading. At transmission, the probability of the recipient to be superspreading is $f_{ss} = \beta_{S,S} / (\beta_{S,S} + \beta_{S,N})$, the fraction of superspreading individuals at equilibrium. We consider that both I_S and I_N populations are otherwise indistinguishable, that is, both populations share the same infectious period ($1/\gamma$)[5,10,11]. The model thus has six parameters, but only five need to be estimated to fully define the model[5,10]. For simulations, we chose parameters of epidemiological interest for re-parameterization: basic reproduction number R_0), infectious period $1/\gamma$, f_{SS}, X_{ss} and sampling probability *s*. In our simulations, we used uniform distributions for these 5 parameters, just as with BD and BDEI (**Supplementary Tab. 4**).

SIMULATIONS

For the parameters R_0, $1/\gamma$, and *s*, that are common to all three birth-death models, the same value boundaries were used across all models (**Supplementary Tab. 4**). We considered two spans of tree size: 'small trees' with 50 to 199 tips and 'large trees' with 200 to 500 tips. We then sampled parameter values uniformly at random within these parameter boundaries with standard Latin-hypercube sampling[60] using PyDOE package. We created 3,990,000 parameter sets for training, 10,000 for validation and early stopping, another 10,000 for testing parameter inference and model selection (comparison with BEAST2 used a subset of 100, for computing time reasons), and 1,000,000 parameter sets for fast computation of CIs.

With these parameter sets, we simulated trees under each birth-death model using our implementation in Python of Gillespie algorithm[61], based on a standard forward simulator. Comparable accuracies (as in **Fig. 3** and **Supplementary Fig. 1**, both for BEAST2 and our methods) were reached on test simulations obtained with a well-established (but slower) simulator: TreeSim[4,5,7] (data not shown).

Each simulation started with one infectious individual (the class was chosen randomly under the BDSS model) and stopped when we obtained a tree with the given number of sampled individuals (tips). If the epidemic died away stochastically, that is, there was no more infectious tips left due to stochastic death before reaching the given tree size, we re-initialized the simulation up to 100 times. Only around 11% of simulations reached more than 2 iterations (20% for BDSS), and less than 0.5% reached more than 50 iterations for all models. If still no tree of given size was obtained after 100 iterations, we discarded the parameter set (less than 0.3% of all sets) and generated a new one to keep the desired number of simulations. This enabled us to maintain a nearly uniform coverage of parameter space, within selected parameter boundaries.

We simulated 100 'huge' trees for each model, with the same parameter values as for the 100 large trees used for testing (Fig. 3). These trees were simulated with treesimulator (https://pypi.org/project/treesimulator). The Snakemake[62] pipeline for huge trees simulation along with the simulated trees are available on GitHub.

METHOD COMPARISON

Parameter inference with BEAST2

To assess the accuracy of our methods, we compared it with a well-established Bayesian method, as implemented in BEAST2 (version 2.6.2). We used the BDSKY package[4] (version 1.4.5) to estimate the parameter values of BD simulations and the package bdmm[12,13] (version 1.0) to infer the parameter values of BDEI and BDSS. Furthermore, for the inference on BDSS simulations, instead of BEAST 2.6.2 we used the BEAST2 code up to the commit nr2311ba7, which includes important fixes to operators critical for our analyses. We set the Markov Chain Monte Carlo (MCMC) length to 5 million steps for the BD model, and to 10 million steps for the BDEI and BDSS models.

The sampling probability was fixed during the estimation. Since the BD, BDEI and BDSS models implemented in BEAST2 do not use the same parametrizations as our methods, we needed to apply parameter conversions for setting the priors for BEAST2 inference (**Supplementary Tab. 6**), and for translating the BEAST2 results back to parameterizations used in our methods, in order to enable proper comparison of the results. More specifically, the BEAST2 parameters can be converted to those used in our methods, that is, instead of infectious period and incubation period, BEAST2 uses the inverse of these, namely the infectious rate and incubation rate, respectively; instead of

superspreading transmission ratio and superspreading fraction at equilibrium, it uses individual sub-component parameters $R_{0,SS}$, $R_{0,SN}$, $R_{0,NS}$ and $R_{0,NN}$, which we will collectively refer to as "partial R_0". For BDSS, the BEAST2 prior was thus not the same as that of our simulations for BDSS (**Supplementary Tab. 4, 6**), since BEAST2 does not infer the same parameters. We used the range of all parameter values used in our simulations to set the boundaries of uniform prior distributions of parameters inferred by BEAST2. The initial values in the MCMC were set to the medians observed in the training set. During the inference, the parameter values were constrained in the same way as in the simulations, namely, we used the constraint $R_{0,SS} \times R_{0,NN} = R_{0,SN} \times R_{0,NS}$ (equivalent to $\beta_{S,S} \times \beta_{N,N} = \beta_{S,N} \times \beta_{N,S}$) in the BDSS model inference. Furthermore, the effective frequency of superspreading individuals (parameter called "geo-frequencies" in *bdmm*) was constrained to be between 5% and 20%. Due to the parameter conversions, and despite these constraints the inferred f_{ss} and X_{ss} can reach values outside the boundaries used for simulations, in which case we set them to the closest boundary for fair comparison with deep learning methods in **Fig. 3** (*e.g.*, if the median *a posteriori* f_{ss} was estimated to be larger than 0.20, it was set to 0.20 and if inferred f_{ss} was less than 0.05, it was set to 0.05). The goal of this correction was to avoid penalizing BEAST2 when it converged to local minima outside of the parameter boundaries used for simulations, which are implicitly known to NNs since they were trained on simulations with parameters within these boundaries.

After we obtained the parameters of interest from the original parameters estimated by BEAST2, we evaluated the Effective Sample Size (ESS) on all parameters. We reported the absolute percentage error of the median of *a posteriori* values, corresponding to all reported steps (reported steps being spaced by 1,000 actual MCMC steps) past the 10% burn-in. For simulations for which BEAST2 did not converge, we considered the median of the parameter distribution used for simulations (**Fig. 3, Supplementary Tab. 1-2, Supplementary Fig. 2**) or excluded them from the comparison (**Supplementary Tab. 1-2**, values reported in brackets, **Supplementary Tab. 5**).

For the HIV application, the prior of infectious period was set to [0.1, 30] years (uniform). All the other parameters had the same prior distributions as used in simulations and shown in **Supplementary Tab. 4, 6.**

Model selection with BEAST2

We performed model selection under BEAST2 using Akaike's information criterion through MCMC (AICM)[32,33]. The AICM is based on the following formula:

$$AICM = 2s_l^2 - 2l$$

where l and s_l^2 are the sample mean and variance of the posterior log-likelihoods. The AICM is an equivalent of AIC and the model with lowest AICM value is selected.

For 100 simulations obtained with each model (BD, BDEI and BDSS for large trees, BD and BDEI for small trees), we performed parameter estimation with BEAST2 under each model, computed AICM considering the whole MCMC, but excluding 10% burn-in (*i.e.*, 9,000 log-likelihood values for BDEI and BDSS considered in total, 4,500 for BD). The results of model selection are shown in **Supplementary Tab. 5**. The BDEI and BDSS simulations for which BEAST2 did not reach an ESS of 200 for all parameters were excluded from the computation of model selection accuracy for all methods.

Linear regression

For each model, linear regression was trained using reduced and centered summary statistics (using scikit-learn package, as with FFNN). Its bias and accuracy were assessed using the same criteria as for the NN approaches (**Supplementary Tab. 1-2, Supplementary Fig. 6**).

FFNN-CBLV

We trained an FFNN on CBLV representation. The FFNN architecture was close to the one described in ***Architecture*** with one extra hidden layer, so 5 layers in total, organized in a funnel shape with 128-64-32-16-8 neurons and 1 output layer of size 2-4 depending on the number of parameters to be estimated. The setting during the training and the sizes of training, validation and testing sets were the same as for the CNN-CBLV. Its bias and accuracy were assessed using the same criteria as for other NN approaches (**Supplementary Tab. 1-2, Supplementary Fig. 5**).

TreePar

We used TreePar[5] for MLE. With BD, we obtained results close to estimates under BEAST2, which is consistent with former studies. TreePar[5] uses an exact analytical formula of likelihood for BD and thus these (and BEAST2) results are theoretically optimal.

We also performed several trials to do parameter inference for the more complex models (*i.e.*, BDEI and BDSS), but in a large number of cases, we encountered numerical problems (*e.g.*, underflow or overflow issues), which resulted in infinite negative log-likelihood values, and eventually failed runs. When the calculations did not fail, we found that many estimations under BDSS and BDEI had lower likelihood than estimations performed with (nested) BD on the same input data. These numerical issues were confirmed by the authors of the TreePar package, with no solution available at the moment.

Null models

To assess how much information was learned on given problem, we compared FFNN-SS and CNN-CBLV to two null models.

The first null model was the FFNN trained for each model on 4,000,000 simulations using SS, but with randomly permuted target values (*i.e.*, the initial correspondence between the SS and underlying parameter values was lost, while the range of values was conserved). We then predicted parameters for 10,000 test simulations (100 for comparison with BEAST2) and measured the mean absolute relative error (MRE; **Supplementary Tab. 1**). In such a case, the FFNN always predicted values close to the value with the lowest value of the cost function (*e.g.*, 2.2 for parameter values uniformly sampled between 1 and 5). The MRE of this approach represents the lowest MRE that machine learning approaches can have in the absence of information, but the knowledge of the parameter distribution. This can be used to get an idea of how well the trained approaches perform and how much information regarding each parameter they can extract from the data.

The second null model was a set of random values sampled from the parameter ranges that were used for simulations (**Supplementary Tab. 4**). In this model, as opposed to the previous null model, there is no training phase and we do not learn the best compromise in the absence of information.

PERFORMANCE ASSESSMENT

Mean relative error MRE

To compare the accuracy of parameter estimation, we used 100 simulated trees per model. We computed the mean absolute relative error (MRE, **Fig. 3-4, Supplementary Tab. 1, Supplementary Fig. 2-4**) between (1) the true (or

target) parameter values and the predicted values for machine learning approaches; and (2) the true (or target) parameter values and the median *a posteriori* values obtained with BEAST2, which are more stable and accurate than maximum *a posteriori* values:

$$MRE = \frac{1}{100}\sum_{i=1}^{100}\frac{|predicted_i - target_i|}{target_i}.$$

We plotted individual absolute relative errors (RE) of predictions (**Fig. 3-4**, **Supplementary Tab. 1, Supplementary Fig. 2, 4**) for each simulation *i*, calculated as:

$$RE_i = \frac{|(predicted_i - target_i)|}{target_i}$$

Not being limited by the computational cost for machine learning approaches, we computed the same metric but on 10,000 simulations (**Supplementary Fig. 3, 5-6;** results from 1,000 simulations plotted in **Supplementary Fig. 7**).

We assessed the statistical significance of MRE differences using two-sided paired z-test. The two NN approaches were also compared using the same test, but no significant differences were found.

Mean relative bias MRB

To compare the bias in parameter estimation, we used 100 simulated trees per model. We computed the mean relative bias (MRB) between (1) the true (or target) parameter values and the predicted values for machine learning approaches; and (2) the true (or target) parameter values and the median *a posteriori* values obtained with BEAST2 (**Supplementary Tab. 2**):

$$MRB = \frac{1}{100}\sum_{i=1}^{100}\frac{(predicted_i - target_i)}{target_i}.$$

Comparison of likelihood values for sets of parameter estimates obtained with different methods

To assess the performance of different methods, we also studied the likelihood values of parameter estimates obtained with BEAST2, CNN-CBLV and FFNN-SS. For BD, we computed the likelihood using TreePar and compared it to the likelihood value of target parameter values (**Supplementary Tab. 3**).

As TreePar was problematic with BDEI and BDSS (see above), we tried to take on the same approach for BDEI and BDSS with BEAST2, but imposing a single MCMC step. Nevertheless, this did not yield sufficient results to perform

sound comparison, since for example with FFNN-SS predictions, the likelihood was obtained only for 57/100 parameter estimates for BDEI and 49/100 for BDSS. In the remaining cases, BEAST2 either failed to return consistent likelihood values, or was unable to calculate likelihood for the initial parameter values.

Model selection accuracy

We performed model selection with CNN-CBLV, FFNN-SS and BEAST2 on 100 simulations obtained with each model (10,000 for a sub-comparison of CNN-CBLV and FFNN-SS). Results are shown in **Supplementary Tab. 5** in the form of confusion matrices, where the columns represent the true/target classes, and the rows are the predicted classes. We then computed the accuracy of each method:

$$accuracy = \frac{\#\, true\ predictions}{\#\, total\ predictions}.$$

For BEAST2 model selection and large trees, the chain did not converge (displayed as “ESS<200” in **Supplementary Tab. 5**) for 24.3% simulations of large trees and 4.5% simulations of small trees. We did not consider these in accuracy measurements, for all the methods.

Comparison of time efficiency

For FFNN-SS and CNN-CBLV, we reported the average CPU time of encoding a tree (average over 10,000 trees), as reported by NextFlow workflow manager[56], a pipeline software that we used. The inference time itself was negligible.

For BEAST2, we reported the CPU time averaged over 100 analyses with BEAST2 as reported by NextFlow. For the analyses with BDEI and BDSS models, we reported the CPU time to process 10 million MCMC steps, and for the analyses with BD, we reported the CPU time to process 5 million MCMC steps. To account for convergence, we re-calculated the average CPU time considering only those analyses for which the chain converged and an ESS of 200 was reached across all inferred parameters.

The calculations were performed on a computational cluster with CentOS machines and Slurm workload manager. The machines had the following characteristics: 28 cores, 2.4Ghz, 128 GB of RAM. Each of our jobs (simulation of one tree, tree encoding, BEAST2 run, etc.) was performed requesting one CPU core. The neural network training was performed on a GPU cluster with Nvidia Titan X GPUs.

HIV DATASET

We used the original phylogenetic tree reconstructed by Rasmussen *et al.*[25] from 200 sequences corresponding to the largest cluster of HIV-infected men-having-sex-with-men (MSM) subpopulation in Zurich, collected as a part of the Swiss Cohort Study[24]. For details on tree reconstruction, please refer to their article.

PHYLODEEP SOFTWARE

FFNN-SS and CNN-CBLV parameter inference, model selection, 95% CI computation and *a priori* checks are implemented in the PhyloDeep software, which is available on GitHub (github.com/evolbioinfo/phylodeep), PyPi (pypi.org/project/phylodeep) and Docker Hub (hub.docker.com/r/evolbioinfo/phylodeep). It can be run as a command-line program, Python3 package and a Docker container. PhyloDeep covers the parameter subspace as described in **Supplementary Tab. 4**. The input is a dated phylogenetic tree with at least 50 tips and presumed sampling probability. The output is a PCA plot for *a priori* check, a csv file with all SS, and a csv file with probabilities of each model (for model selection) and point estimates and 95% CI values (for parameter inference with selected model). The installation details and usage examples are available as well on GitHub.

ADDITIONAL REFERENCES

58. Zarestkii, K. Reconstructing a tree from the distances between its leaves. (in Russian) *Uspehi Mathematicheskikh Nauk* **20,** 90-92 (1965).

59. Efron, *B. Breakthroughs In Statistics, Ch. Bootstrap Methods: Another Look at the Jackknife.* (Springer, New York, 1999).

60. McKay, M., Beckman, R., Conover, W. A Comparison of Three Methods for Selecting Values of Input Variables in the Analysis of Output from a Computer Code. *Technometrics* **21,** 239-245 (1979).

61. Gillespie, D.T. Exact stochastic simulation of coupled chemical reactions. *The Journal of Physical Chemistry* **81,** 2340-2361 (1977).

62. Köster, J., Rahmann, S. Snakemake - a scalable bioinformatics workflow engine. Bioinformatics. **28**(19), 2520-2 (2012).
