# Supplementary material for: Deep learning from phylogenies to uncover the epidemiological dynamics of outbreaks
Source: Nat Commun. 2022 Jul 6;13:3896. doi: 10.1038/s41467-022-31511-0 (PMC9258765; doi:10.1038/s41467-022-31511-0)
Supplement: Supplementary file 3 — Reporting Summary [file 41467_2022_31511_MOESM3_ESM.pdf]

nature portfolio

Corresponding author(s): J. Voznica, A. Zhukova, O. Gascuel

Last updated by author(s): 2022-6-20

Reporting Summary

Nature Portfolio wishes to improve the reproducibility of the work that we publish. This form provides structure for consistency and transparency in reporting. For further information on Nature Portfolio policies, see our Editorial Policies and the Editorial Policy Checklist.

Statistics

For all statistical analyses, confirm that the following items are present in the figure legend, table legend, main text, or Methods section.

n/a	Confirmed	
☐	☒	The exact sample size (n) for each experimental group/condition, given as a discrete number and unit of measurement
☐	☒	A statement on whether measurements were taken from distinct samples or whether the same sample was measured repeatedly
☐	☒	The statistical test(s) used AND whether they are one- or two-sided<br>*Only common tests should be described solely by name; describe more complex techniques in the Methods section.*
☒	☐	A description of all covariates tested
☒	☐	A description of any assumptions or corrections, such as tests of normality and adjustment for multiple comparisons
☐	☒	A full description of the statistical parameters including central tendency (e.g. means) or other basic estimates (e.g. regression coefficient) AND variation (e.g. standard deviation) or associated estimates of uncertainty (e.g. confidence intervals)
☐	☒	For null hypothesis testing, the test statistic (e.g. F, t, r) with confidence intervals, effect sizes, degrees of freedom and P value noted<br>*Give P values as exact values whenever suitable.*
☐	☒	For Bayesian analysis, information on the choice of priors and Markov chain Monte Carlo settings
☒	☐	For hierarchical and complex designs, identification of the appropriate level for tests and full reporting of outcomes
☐	☒	Estimates of effect sizes (e.g. Cohen's d, Pearson's r), indicating how they were calculated

Our web collection on statistics for biologists contains articles on many of the points above.

Software and code

Policy information about availability of computer code

Data collection	We use a phylogenetic tree reconstructed from sequences of HIV patients from Zurich, for a showcase application of our methods. This tree was published in "Phylodynamics on local sexual contact networks" by David A Rasmussen et al, 2017, PloS Computational Biology and made publicly available on github: https://github.com/davidrasm/PairTree (all confidential information has been removed).<br>Simulated data was obtained using our own simulator in Python 3.6 available at https://github.com/evolbioinfo/phylodeep/tree/main/Data_publication together with all testing trees and estimates provided by our methods and by BEAST2.
Data analysis	The PhyloDeep package (version 0.3) is under the GPL v3.0 license and uses Python (3.6) and Python libraries : ete3 (version 3.1.2 under GNU general licence); pandas (version 1.1.5); numpy (version 1.19.5); scipy (version 1.1.0); scikit-learn (version 0.19.1); tensorflow (version 1.13.1); joblib (version 0.13.2); h5py (version 2.10.0); Keras (version 2.4.3 under Apache 2.0 license); matplotlib (version 3.1.3 under PSF license).<br><br>We provide (i) the source code of PhyloDeep, (ii) the code of the tree simulators used to train the deep learners and (iii) the log files obtained with BEAST2 on GitHub (github.com/evolbioinfo/phylodeep). The code has been deposited in Zenodo (https://doi.org/10.5281/zenodo.6646668).<br><br>We used the version 2.6.2 of BEAST2 for BD and BDEI inferences and BEAST2 compiled up to the commit nr2311ba7 for BDSS inferences, and version 3.3 of TreePar. For BEAST2 inferences, we used BEAST2 libraries bdmm (version 1.0) and BDSKY (version 1.4.5).<br><br>We used Snakemake (version 5.10.0) and Nextflow (version 21.04.3.5560) pipeline managers for simulations and analyses of simulated data.

For manuscripts utilizing custom algorithms or software that are central to the research but not yet described in published literature, software must be made available to editors and reviewers. We strongly encourage code deposition in a community repository (e.g. GitHub). See the Nature Portfolio guidelines for submitting code & software for further information.

Data

Policy information about availability of data

All manuscripts must include a data availability statement. This statement should provide the following information, where applicable:

- Accession codes, unique identifiers, or web links for publicly available datasets
- A description of any restrictions on data availability
- For clinical datasets or third party data, please ensure that the statement adheres to our policy

The data generated in this study (simulated trees, BEAST2 logs, and the results of BEAST2 and PhyloDeep runs), as well as the HIV phylogenetic tree for Zurich epidemic (a showcase application) are provided on GitHub (github.com/evolbioinfo/phylodeep, version 0.3) and have been deposited in the Zenodo database under accession code https://doi.org/10.5281/zenodo.6646668. The simulated trees were obtained with our simulator (see Code availability), the HIV tree was previously published by Rasmussen et al, PloS Computational Biology 2017, and is available on their GitHub: github.com/davidrasm/PairTree (all confidential information has been removed).

Human research participants

Policy information about studies involving human research participants and Sex and Gender in Research.

Reporting on sex and gender	*Use the terms sex (biological attribute) and gender (shaped by social and cultural circumstances) carefully in order to avoid confusing both terms. Indicate if findings apply to only one sex or gender; describe whether sex and gender were considered in study design whether sex and/or gender was determined based on self-reporting or assigned and methods used. Provide in the source data disaggregated sex and gender data where this information has been collected, and consent has been obtained for sharing of individual-level data; provide overall numbers in this Reporting Summary. Please state if this information has not been collected. Report sex- and gender-based analyses where performed, justify reasons for lack of sex- and gender-based analysis.*
Population characteristics	*Describe the covariate-relevant population characteristics of the human research participants (e.g. age, genotypic information, past and current diagnosis and treatment categories). If you filled out the behavioural & social sciences study design questions and have nothing to add here, write "See above."*
Recruitment	*Describe how participants were recruited. Outline any potential self-selection bias or other biases that may be present and how these are likely to impact results.*
Ethics oversight	*Identify the organization(s) that approved the study protocol.*

Note that full information on the approval of the study protocol must also be provided in the manuscript.

Field-specific reporting

Please select the one below that is the best fit for your research. If you are not sure, read the appropriate sections before making your selection.

☒ Life sciences ☐ Behavioural & social sciences ☐ Ecological, evolutionary & environmental sciences

For a reference copy of the document with all sections, see nature.com/documents/nr-reporting-summary-flat.pdf

Life sciences study design

All studies must disclose on these points even when the disclosure is negative.

Sample size	We assessed the accuracy of our methods with the state-of-the art methods on 100 simulations per model and per condition. The number was determined by the computational time consumption of the state-of-the art method BEAST2 (between 5,700 and 7,900 CPU hours for the inference on 100 samples for complex models).<br><br>Not being limited by the inference speed of our methods, we also provide accuracy assessment on 10,000 simulations per model and per condition.<br><br>For the showcase application, we performed analysis on a previously analyzed and published dated phylogeny reconstructed from 200 sequences of HIV patients from Zurich.
Data exclusions	For simulations (for training, validation and test sets) we excluded simulations that could not reach the given tree size after 100 trials. It was because the simulated tree became extinct before reaching the wished size. This represents less than 0.3% simulations in total. We replaced these unsuccessful combinations of parameters with new randomly generated ones, to reach the desired number of simulated trees.
Replication	All of our results are fully reproducible using the programs, pipelines and test sets (50,000 test samples in total) available from Github.
Randomization	NA. There are no "experimental groups", "allocations" or "covariates" associated with the HIV sequences we used to illustrate our methods.

	For the other simulation-based experiments, we used independent training and test sets.
Blinding	NA. There are no "experimental groups," "allocation" or "investigators" in this study, which is entirely computational.

Reporting for specific materials, systems and methods

We require information from authors about some types of materials, experimental systems and methods used in many studies. Here, indicate whether each material, system or method listed is relevant to your study. If you are not sure if a list item applies to your research, read the appropriate section before selecting a response.

Materials & experimental systems

n/a	Involved in the study
☒	☐ Antibodies
☒	☐ Eukaryotic cell lines
☒	☐ Palaeontology and archaeology
☒	☐ Animals and other organisms
☒	☐ Clinical data
☒	☐ Dual use research of concern

Methods

n/a	Involved in the study
☒	☐ ChIP-seq
☒	☐ Flow cytometry
☒	☐ MRI-based neuroimaging
